# Subjects to Be Treated

**Published:** 1854-01

**Authors:** 


The subjects presented from time to time will be such as

How to eat.

How to sleep.

How to exercise.

How to dress.

How to walk.

How to read in public.

How to declaim with ease and fluency.

How to select food so as to make it both nutritive and medicinal.

How persons become dyspeptic.

How and why the health of the young so often begins to fail while pursuing an education.

How they may so conduct their studies as to preserve the health, and yet accomplish in the course of the year a larger amount of mental and literary labor.

If an essay is admitted, it will be among others from eminent practical dentists, as to the best means of preserving the teeth perfect to the close of life, as perfect teeth are essential to a distinct enunciation, which is of indispensable importance to public men, whether professional or literary.

I will endeavor to make this publication such an one as will be proper for the youth of both sexes to read with increasing interest and attention, so as to induce them early, while the constitution is yet vigorous and unimpaired, so to study the nature and effects, on the human frame, of food, clothing, air, exercise, cheerfulness, system, industry, profitable and interesting employment, as will be an effectual guard against those negligences and indiscretions which so often lay the foundation for an early grave and the utter blasting of parental hope; for none but an affectionate parent can ever know the abiding anguish which rends the heart, to the last day of life, the remembrance of a son or daughter early dead, and I know that such anguish could be prevented in multitudes of hearts, if the principles were early inculcated, which this Journal will advocate from time to time; and my hope is, that the father or the mother of a family will not only take a copy for their own use and constant reference, but will also order a copy for one or more of their children, which, from the fact of its being their own, will secure their personal interest in it, which cannot be done so well, when taken for the whole family.

